# A Reinforcement Sensitivity Theory of Violent Extremist Propaganda: The Motivational Pathways Underlying Movement Toward and Away From Violent Extremist Action

**DOI:** 10.3389/fpsyg.2022.858392

**Published:** 2022-05-19

**Authors:** Neil Shortland, Jill Portnoy, Presley McGarry, Arie Perliger, Thomas Gordon, Natalie Anastasio

**Affiliations:** School of Criminology and Justice Studies, University of Massachusetts Lowell, Lowell, MA, United States

**Keywords:** Reinforcement Sensitivity Theory, online radicalization, physiology, BIS/BAS, media

## Abstract

Anecdotal evidence supports than engaging with violent extremist content online facilitates the radicalization process. However, there is a consistent lack of empirically grounded research to provide insight into the psychological process through which this influence occurs (if at all). As such, most theories often fail to accommodate both the multifinality (the concept that many people are exposed to violent extremist material, yet never engage in violent extremism), and equifinality (the concept that people can view a range violent extremist content, yet all end up engaging in violent extremism) that naturally is observed in those who engage with violent extremist content online and those who engage in violent extremist behavior. This paper presents Reinforcement Sensitivity Theory (RST) as a theoretical framework to inform understanding of the process that governs the interaction between violent extremist material online and engaging with violent extremism. RST is a motivational theory which has been applied to a range of benevolent and deviant behaviors. Specifically, we argue that RST is suitable to explain the effect of violent extremist content online because (1) it outlines multiple differentiated motivational pathways that can account for multifinality and equifinality observed in those who engage in violent extremist behavior and (2) the extant neurological and psychophysiological research using RST provides a empirically supported framework for developing both research methods and verifiable hypotheses to advance our understanding of how, if at all, violent extremist content online contributes to the process of radicalization.

## Introduction

The use of the Internet by violent extremists has become a primary focus for academic research (Neo, 2019). Extremist organizations currently use information technology, specifically the Internet, as a platform to recruit, disseminate ideological messages, deliver threats, release instructional materials to facilitate the actions of others, as well as to plan and coordinate violent extremist attacks (Weimann, 2006). The Internet plays a central role in facilitating the processes through which Western individuals support or join extremist groups by facilitating contact and planning between would-be-recruits and recruiters, and extremist material on the Internet has also directly facilitated attempts to, or inspired the perpetration of domestic attacks (see Lemieux et al., 2014)^1^. Over the past two decades anecdotal evidence supported a growing assertion that individuals who engage in acts of violent extremism have, at varying points, in varying ways, and to varying degrees, engaged with violent extremist content online (see Conway, 2006; Hoffman, 2006; Freiburger and Crane, 2008; Bowman-Grieve, 2009; Weimann, 2011; Bowman-Grieve and Conway, 2012; Holbrook et al., 2013; Von Behr et al., 2013; Ekman, 2014). Despite this, from a theoretical perspective “the reality […] is that insufficient substantive empirically grounded social science research has been undertaken to date in order to allow us to convincingly answer [the question whether the internet is influential]” (Conway, 2017, p. 82; Frissen, 2021).

There are several pre-existing issues that have hindered the development and testing of theories pertaining to the link between violent extremist content online and violent extremist action. Firstly, in the psychological study of extremism, as a whole, there has been a significant lack of progress in conceptualizing the radicalization process (see Sageman, 2014) with efforts hampered by little/no primary source data (Silke, 2000, 2004; Schuurman, 2019). Secondly, as highlighted by Horgan (2019), most theories often fail to accommodate the issues of multifinality (the concept that people can experience the same life events or have similar histories, yet their developmental outcomes can vary widely; Howe, 2011), and equifinality (the principle that in open systems a given end state can be reached by many potential means; Gill et al., 2021). Meaning, that theories lack pathways of differentiation that can explain why, while many people are exposed to violent extremist content, only a few progress down a pathway of radicalization. Furthermore, many people end up at a state of “being radicalized” but come from a range of diverse exposures to violent extremist content online (Horgan J. et al., 2016; Horgan J. G. et al., 2016). Despite this, a range of experimental research has shown that the effect of exposure to violent extremist content online depends on the personality of the viewer (Shortland et al., 2017, 2020). These issues, coupled with the widely espoused view that personality is critical to the radicalization process (e.g., McGregor et al., 2015), supports the need for explanations for the role of violent extremist content to (at least partly) focus on the role that individual differences in personality play on core motivations to engage in violent extremist action.

### Reinforcement Sensitivity Theory

RST presupposes that individual differences in responses to stimuli stem from different sensitivities of basic brain systems that respond to novel, punishing, and reinforcing stimuli (Gray, 1973, 1982). The original version of RST (Gray, 1982) proposed a reward system (the behavioral activation system, BAS), punishment system (the behavioral inhibition system, BIS), and threat-response system (Fight/Flight system, FFS). Activation of the BAS system was posited to promote approach behavior and positive affect, while the activation of the BIS system was thought to promote withdrawal behavior and negative affect (Smillie et al., 2006). After a wave of early empirical research, many tenants of RST were not validated, leading to refinements of the theory in 2000 (Gray and McNaughton, 2000; see Pickering and Gray, 1999; Corr, 2001, 2004; Jackson, 2003). This refined model of RST altered the underlying relationship between the three systems. The BAS still functions as a reward system, while the FFS was renamed the FFFS (the additional “F” representing “freeze”). This model also expanded the role of the BIS as not simply a response to conditioned negative responses, but also to monitor and respond to motivational conflict that emerges when *both* the BAS and FFFS are activated. The BIS is thus no longer considered a punishment system (this function was moved to the FFFS) but is instead an anxiety-biased moderator between the BAS and FFFS (Gray and McNaughton, 2000). In the updated version of RST, stimuli perceived as positive activate the BAS and motivate approach behaviors toward target reinforcers, or goals, while stimuli perceived as negative activate the FFFS, motivating the individual to avoid potential threats. The BIS coordinates the response by attempting to resolve conflicting inputs when a stimulus activates both BAS and FFFS (Gray and McNaughton, 2000). Gray’s theory has been linked with primary psychopathy (Ross et al., 2007), aggressive inclinations (Harmon-Jones and Peterson, 2008), state anger (Carver, 2004) and general aggressive cognitions (Putman et al., 2004). It has also been applied to voter behavior after the effects of experiencing terrorism (Marcus and MacKuen, 1993).

**FIGURE 1:**
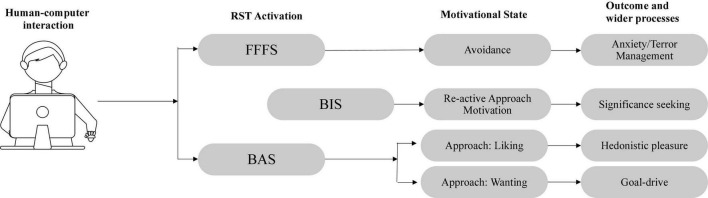
Four motivational pathways toward and away from violent extremist action.

The application of RST to violent extremist material online in this article allows us to advance a preliminary theoretical model to that proposes four possible pathways that occur based on the interaction of an individual with violent extremis content online (see Figure 1). Each pathway a range of predictive personality traits that are associated with each pathway. Furthermore, each pathway does, to varying degrees, reflect extant theories of the psychology of violent extremism as well as, integrate with previous research on the correlates of RST functioning in a range of domains. What this model provides is an integration of decades of research on RST and violent extremism that allows us to how the interaction of individual state and trait functions manifest in quantifiably different RST activation states which, crucially, imply different forms of motivation toward (or away) from violent extremism in general. Leveraging the extant research on RST and violent extremism, we outline RST, and each of these pathways below.

## Four Motivational Pathways Toward and Away From Violent Extremist Action

### The FFFS: Avoidance as a Response to Violent Extremist Content Online

Many individuals who are exposed to violent extremist content online are repelled by the content (Shortland et al., 2017), and this is the first possible outcome pathway: an individual is being repelled from the material, driven by FFFS activation. The role of the BIS/FFFS in *repelling* people from terrorism is not a new assertion. Terrorism, has, after all been termed the “Politics of Fear” (Pyszczynski, 2004). BIS and FFFS processes include behavioral inhibition, driven by fear responses, trait anxiety, and threat avoidance (Gray and McNaughton, 2000; McNaughton and Corr, 2004; Perkins et al., 2007), all of which are common reactions to acts of extremism or extremist propaganda. Terror Management Theory (TMT) posits that exposure to threatening materials reminds individuals of their own mortality, creating an in-group bias involving increased pro-social behavior and empathy for the members of their in-group (Hewstone et al., 2002; Schimel et al., 2006). Accordingly, exposure to terrorist attacks is associated with increases in fear and anxiety (Marshall et al., 2007; Toohey and Taylor, 2008; Aly and Green, 2010). Consistent with this, television viewing of rebroadcast visuals of 9/11 was found to increase fear and anxiety (Fahmy et al., 2006). Anxiety is also higher in adults who directly experienced loss or witnessed a terrorist event or are exposed to televised terrorism-related material (Schuster et al., 2001; Schlenger et al., 2002). Children are equally affected by news coverage of 9/11, regardless of whether they have witnessed a terrorist attack (Pfefferbaum et al., 2001). Taken together, this body of research suggests that many individuals experience an FFFS response when exposed to extremist material which causes fear, anxiety, and a motivation to withdraw from the material.

### The Behavioral Activation System: Approach Motivation as a Response to Violent Extremist Content Online

The primary function of the BAS is to move an organism from a start state (e.g., hunger) through acquisition and toward the final biological reinforcer, or goal (e.g., eating; Corr, 2013; Corr and Krupić, 2017). Individual differences in BAS processing have been found to relate to individual differences in approach-related personality traits and behavior (Gray and McNaughton, 2003; Segarra et al., 2014). Traditional BAS traits have been previously identified as possible risk factors for engagement in violent extremism (facilitated *via* both online and offline radicalization). Extraversion and impulsivity have been viewed as manifestations of BAS dimensions (Depue and Collins, 1999), both of which are thought to be personality traits associated with extremism. Süllwold (1981), found that increased extraversion among Red Army Faction members predisposed these youth to join terrorist movements (also see Canadian Network for Research on Terrorism, Security and Society [TSAS], 2015). Sensation seeking is also a BAS trait that psychologists have argued predisposes an individual to the processes leading to terror-related involvement (Silke, 2008). Researchers have claimed that youth become radicalized because of the “seductive” and “adventurous” dimensions of extremist groups (Atran, 2008). BAS activation is also associated with sensation and novelty seeking (Franken and Muris, 2006; Segarra et al., 2014). Along with sensation seeking (Silke, 2003), risk tolerance has also been suggested as a risk factor for terrorism (Crenshaw, 1981). Further, while BIS sensitivity predicted fear reactions after the 11 September attacks, BAS sensitivity was linked to anger responses and motivational drive (Carver, 2004) and extraversion (a BAS trait) correlates negatively with terrorism anxiety (Hawi et al., 2019). Finally, general criminality is a well-known risk factor for involvement in extremism (LaFree et al., 2018) and higher scores on assessments of trait-BAS sensitivity are associated with an individual having more frequent arrests (Taylor and Eitle, 2015). Furthermore, physiological pathways associated with BAS functioning are also linked to testosterone, social dominance, and social aggression—all of which have been postulated as risk factors related to extremist behavior (Levin et al., 2003; Caluya, 2013; Möller-Leimkühler, 2018).

One of the more recent focusses in the application of RST is disaggregating the nature of BAS activation, and the implications of this for behavior. In a review of the five most frequently used RST questionnaires, Krupić et al. (2016) classified the BAS scales from the five questionnaires into four groups that represented different forms of BAS activation: wanting, striving, liking, and capturing—these constructs then shaped the latest version of the RST-Personality Questionnaire (RST-PQ; Corr and Cooper, 2016) which separates the BAS into four interrelated processes: Reward Interest, Goal-Drive Persistence, Reward Reactivity, and Impulsivity. Each of these inter-related processes reflect a different type of approach behavior. Reward Interest represents the first stage of an approach motivation and the search for a new reward. Goal-Drive Persistence measures an individuals’ degree of persistence toward achieving that goal. Reward Reactivity measures emotional reactivity to reward and Impulsivity reflects the fast reaction at the final stage of an approach process to capture the reward. This reflects the known multidimensionality of reward systems. Berridge and Robinson (2003) introduced three components of reward system: learning, liking, and wanting to represent the difference between motivational aspects of reward (wanting something) and affective reactions to a reward. In a recent effort to unify and integrate the many different labels used to represent the same underlying BAS construct, Krupić and Corr (2017) introduced a multidimensional model of approach behavior in which they identified each of the four approach processes. In addition to this they identified the neurobiological process that underpins each form of approach. Within the study of “the terrorist” there have been long-standing calls of the need to disaggregate both the types of individual that engage in extremist behavior (see Gill and Corner, 2013) and the psychological meaning that an individual derives from their engagement (Horgan J. et al., 2016). In disaggregating approach behaviors *via* a multidimensional model of BAS here, we can separate the nature of approach behavior caused in response to exposure to extremist material and the ensuring implications of how, if at all, the nature of this reaction changes over time if at all. Within the current stats of RST research, there is evidence that “wanting” and “liking” are dissociable psychologically and neurologically, with each also based on different underlying physiological pathways (Berridge et al., 2009). While “wanting” is defined by the establishment of declarative goals and the expectations of future outcomes, “liking” is based positive hedonic impact, or pleasure of reward driven by opioid circuits associated with pleasure and immediate gratification (Berridge and Robinson, 2003). As defined by Krupić and Corr (2017), *wanting* is necessary for attaining a reward, while *liking* reflects an individual’s reaction after obtaining a desired reward. While in many cases we want what we like, we can also like a stimulus, but not want to explicitly set goals to acquire it. We apply this dissociation to violent extremism below.

#### Behavioral Activation System Liking

The term “liking” refers to the positive hedonic impact, or pleasure of reward (Berridge and Kringelbach, 2008). Where the word liking in the everyday sense refers to conscious, subjective experience of pleasure, core “liking” response represents the hedonic reaction to reward, regardless of any conscious feelings of pleasure, and is used to describe the objective emotional consequences of reward (Berridge and Robinson, 2003; Berridge and Kringelbach, 2008; Berridge, 2009). It is important to note that “liking” is purely an affective state, and no incentive target or motivation for further reward is needed to trigger hedonic “liking” (Berridge, 2009). Hedonic brain systems process and label sensations with a hedonic valence (the hedonic niceness or nastiness of a stimulus) marking that sensation as pleasurable or “liked” (Berridge and Kringelbach, 2008). Reflecting a positive hedonic valence, the pleasure of a reward elicits an affective “liking” response, which is then experienced as conscious pleasure (Berridge and Kringelbach, 2008). For example, the pleasure of sweetness is generated by the hedonic brain systems that associate pleasure with the sensation of tasting something sweet.

Scales measuring BAS sensitivity include a measure of “liking” that has been associated with reward responsiveness and reward reactivity. These scales are characterized by positive emotionality, and it is thought that this is indicative of a lower threshold of reward value needed to elicit psychological reactions to stimuli. “Liking” has also been associated with trait extraversion and agreeableness (Krupić and Corr, 2017). Further, in a study on engagement with COVID-19 prevention guidelines, approach-related liking has been associated with increased likelihood of engaging in social distancing (Krupić et al., 2021). This hedonic “liking” has distinct neural mechanisms, and objective physiological markers in the brain, as well as objective behavioral effects and subjective emotional effects (Berridge, 2009). In both humans and other animals, affective facial expressions caused by a hedonic response to sweet tastes is a commonly used “liking” measure of pleasure (Berridge and Robinson, 2003, 2016; Berridge and Kringelbach, 2008; Berridge, 2009). Sweet tastes elicit a positive facial response indicating “liking,” while bitter tastes elicit negative facial expressions, indicating “disliking” (Berridge, 2009). “Liking” is thought to involve opioid neurotransmission, and injections of opioid agonists have been found to increase facial “liking” reactions to sweet tastes (Berridge and Robinson, 2003; Berridge, 2009). Research suggests that opioid receptor activation can increase “liking” for certain foods and stimulate appetite (Berridge, 2009). It is thought that the activation of these opioid receptors can enhance the hedonic pleasure valence attributed to a reward, such as sweetness (Berridge, 2009). Additionally, even though typically associated with reward, dopamine depletion has been found to have no effect on “liking” reactions to pleasure (Berridge and Kringelbach, 2008; Berridge and Robinson, 2016). Thus, it is the opioid system that is thought to be more related to pleasure than dopamine systems (Berridge and Robinson, 2003). Dopamine is thought to have an alternative role related to “wanting.”

So, from the perspective of engaging with violent extremist content online, a “liking” response would be precisely that; liking the material and gaining a hedonistic pleasure from it, but with no necessary concurrent activation of a motivational state to acquire (or engage) with the goals being depicted in the media. In terms of access to violent extremist content online, it is viable to propose that many individuals engaging with such content are driven by an immediate liking and opioid-driven reward system without activating concurrent BAS motivations to acquire or engage with such material in the real world. Specifically, an overwhelming number of individuals currently engaging with violent extremist content grossly outweighs the number of individuals who actively seek to be involved with violent extremism (Shortland and Forest, 2020). A video depicting the beheading of Nick Berg by Islamic extremists in Iraq was downloaded over 15 million times, with sites hosting the video receiving over 60,000 hits per hour (Talbot, 2005). Between September and December 2014, 46,000 Twitter accounts were linked to ISIL supporters (Berger and Morgan, 2015). To put this point in perspective, there are currently over 1,000 open terrorist investigations in the United States pertaining to potential Islamic State (ISIS) offenders (Wray, 2018), yet there are only around 40 ISIS-related arrests in the United States each year (a 4% prevalence; see Vidino and Hughes, 2015). Furthermore, reports of the digital behavior of individuals possessing violent extremist propaganda has also noted that those individuals also possessed an array of other deviant material online such as child pornography (e.g., Ritter, 2020).

In relation to the four BAS process outlined in the RST-Personality Questionnaire (RST-PQ; Corr and Cooper, 2016) scales measuring BAS sensitivity include a measure of “liking” that has been associated with reward responsiveness and reward reactivity. These scales are characterized by positive emotionality, and it is thought that this is indicative of a lower threshold of reward value needed to elicit psychological reactions to stimuli. “Liking” has also been associated with trait extraversion and agreeableness (Krupić and Corr, 2017), traits that are associated with engagement in extremist behavior (Süllwold, 1981; Canadian Network for Research on Terrorism, Security and Society [TSAS], 2015).

#### Behavioral Activation System Wanting

Behavioral activation system wanting on the other hand represents the activation of a motivation drive toward the achievement of a goal. Generally referring to a conscious desire with a declarative goal, the term “wanting” refers to incentive salience, a motivational process that makes stimuli attractive (Berridge and Robinson, 2003; Berridge and Kringelbach, 2008; Berridge, 2009; Berridge and Robinson, 2016). As conceptualized by Krupić and Corr (2017), wanting has been conceptualized into two constructs, where *wanting* (capturing) is having a desire for a certain goal, and *incentive motivation* (wanting) is the motivational pathway toward engaging in those actions. These constructs of wanting are thought to have evolved early in evolution as a form of goal direction, and incentive salience is thought to be a separate process from “liking” to facilitate choosing between competing rewards that are equally “liked” (Berridge and Robinson, 2003). Incentive salience is linked to Pavlovian conditioned stimuli or reward-cues, causing cue-triggered “wanting” for a given reward. Mirroring hedonic “liking,” incentive salience is an implicit and objective motivation process that attributes the sensation of desire to rewards and their cues (Berridge and Robinson, 2003), these cues then become triggers of “wanting” (Berridge, 2009). When “wanting” is attributed to a reward stimulus representation, that stimulus (and its associated reward) gains enhanced motivational value (Berridge and Kringelbach, 2008; Berridge, 2009). Alone, a given stimulus (e.g., food or drugs) and its associated reward, are not intrinsically motivating—incentive salience, or attributed “wanting,” is what is thought to make a stimulus a target of motivation (Berridge and Robinson, 2003). Without “liking,” “wanting” is the sensation of desire without sensory pleasure, and “wanting” does not require conscious understanding of the causal relationship between a reward and the hedonic pleasure outcome (Berridge, 2009). Additionally, “wanting” can be triggered without conscious thought, and excessive incentive salience has been linked to irrational “wanting” for stimuli that are not cognitively desired or “liked” (Berridge and Robinson, 2003, 2016; Berridge, 2009).

In example, non-conscious alteration in consumption behavior has been associated with subliminal exposure to differing facial expressions, changing individuals’ desire for and rating of a beverage, with no reported conscious emotional changes (Berridge and Kringelbach, 2008). Increased dopamine levels have been found to quadruple a rat’s “wanting” for food rewards, with no corresponding changes in pleasure “liking” response (Berridge and Robinson, 2016). Dopamine release is triggered by many pleasant rewards and their associated cues (Berridge, 2009), and is related to better behavioral performance triggered by reward cues (Berridge and Kringelbach, 2008; Berridge, 2009). Additionally, dopamine suppression was found to produce no change in pleasure ratings of drug-related reward, even though it reduced desire to consume more of a drug (Berridge and Robinson, 2016). Irrational wanting behaviors, thought to originate from “wanting” without “liking,” are also associated with the activation of dopamine systems (Berridge, 2009).

Addiction related “wanting” is suggested to occur due to incentive salience creating a motivational compulsion, even if there is no pleasure outcome. Dopamine release meditates this effect, causing *hyper*-reactive “wanting” responses toward addiction cues and contexts that then cause more intense incentive salience (Berridge and Kringelbach, 2008; Berridge and Robinson, 2016). Individuals with other various behavioral addictions (e.g., gambling addiction, binge-eating disorders, and pornography addiction) are also thought to have hyper-reactivity toward cues related to their addiction (Berridge and Robinson, 2016). Although rewards that are “liked” are typically also “wanted,” “wanting” can occur in accordance with, in opposition to, or in the absence of cognitive desires, and without “liking” (Berridge and Robinson, 2003; Berridge, 2009). While research has demonstrated how “liking” and “wanting” can be dissociated, both are necessary for experiencing reward (Berridge, 2009). “Wanting” has also been shown to lead to behavioral changes. Scales measuring BAS sensitivity also include a measure of “wanting” associated with the reward interest, impulsivity BAS processes outlined in the RSTPQ, as well as sensation-seeking (Corr and Cooper, 2016; Krupić and Corr, 2017). Ambitiousness and social dominance are also traits associated with individuals high in “wanting” (Krupić and Corr, 2017). “Wanting” has been found to correlate with fast lifestyle behaviors, and individuals high in “wanting,” tend to favor problem solving strategies with immediate benefits (Krupić and Corr, 2017). Supporting this notion, approach-related wanting was also associated with increased level of concerns regarding the COVID-19 pandemic, and increased likelihood of following COVID-19 prevention guidelines (wearing a mask and/or gloves). Additionally, reflective of a fast lifestyle, the BAS process of “wanting” is associated with trait conscientiousness, extraversion, and social dominance (Krupić and Corr, 2017).

In the case of violent extremist content online, if a wanting response is activated, and the goal prioritized, the individual must then engage in a purposeful effort to achieve this goal (incentive motivation). This process requires delayed gratification, and investment in a further (and distal goal). In such instances, the individuals need to be able to main self-control and discipline, while also gaining suitable positive reinforcement *via* “local highs” (Corr and Krupić, 2017); which are short term forms of positive reinforcement. At the same time, they must not focus overly on these local highs, in favor of the long-term goal. Thus, an individual must be able to engage in this process of purposeful effort, at the same time, receive positive feedback from it, while also not forgoing the overall long-term goal in favor of these short-term “local highs.” An example of this, in the realm of online propaganda, may be an individual who begins the process with the overall long-term goal of joining an extremist group abroad (e.g., the Islamic State), but in the process of incentive motivation, receives significant positive feedback in the forms of social acceptance and positive reinforcement for their online activities and role in the online social networks associated with this group. In such instances, the individual may receive such positive feedback from these “preparatory” activities, that they forgo the ultimate goal of behaviorally engaging in terroristic activity. Especially if these “local highs” address the underlying needs that this individual that drove the individual to originally respond to extremist content with wanting. An alternative perspective, which would be in line with recent behavioral trends in the nature of extremist actions, is that if the individual does not have sufficient self-control to engage in a purposeful quest for the ultimate goal, they may impulsively engage in a short-term action that requires less persistence and behavioral commitment; such as an impulsive mass shooting (which represent 15% of lone actor shootings; Gill et al., 2017). At the end of the process the nature of the hedonistic reaction following attainment of the goal should enhance the learning process, which can then influence valuation of the goal. Research on why people leave extremism supports the role of this valuation process in the continuation of the behavior, in that individuals who have engaged in a purposeful process of becoming involved and engaged in extremist behavior can often begin a process of disengagement if they feel disillusioned with their role and actions and these behaviors no longer produce a positive reinforcement (Horgan and Bjorgo, 2009).

### Behavioral Inhibition System: Reactive Approach Motivation as a Response to Violent Extremist Content Online

Behavioral inhibition system conceptualizes the BIS as a conflict detection system, responsible for inhibiting ongoing behavior when conflict is detected between simultaneously active behavioral goals (Mardaga and Hansenne, 2007; Berkman et al., 2009; Hirsh and Kang, 2016). Thus, in instances in which both BAS and FFFS are activated in response to exposure to a stimulus, the BIS governs these avoidant-activated conflict states. With regards to violent extremism, certain BIS functions are closely associated with feeling of meaningless, threats to one’s identity, and sensitivity to social and societal rejection. All of which have been advocated as potential motivators for engaging with violent extremist activity. The quest for significance (Kruglanski and Orehek, 2011) presents a model of radicalization proposes that involvement in terrorism is a route to achieve personal significance. As such, involvement in terrorism is driven by three general drivers of violent extremism: a need for significance, a narrative that provides a means to achieve significance, and a network of like-minded individuals who make the violence-justifying cognitions perceived as morally acceptable. The theory holds that central to all action is the desire to “be someone,” and to have meaning in one’s life. Kruglanski et al. (2014) found that members of the Liberation Tigers of Tamil Eelam (a militant separatist group fighting for an independent homeland for Hindu Tamils in Northeastern Sri Lanka) reported feelings of shame in the preceding weeks predicted their support and engagement in violent activities. A more recent cross-cultural survey in Indonesia, Morocco and Sri Lanka confirmed the link between quest for significance—particularly collective significance—and support for political violence (Jasko et al., 2019).

Individuals with heightened BIS sensitivity have reported feeling less meaning and purpose in their lives (McGregor et al., 2013). Additionally, individuals with increased BIS sensitivity tend to be more attentive to the negative aspects of a situation and have more pessimistic outlooks (Hirsh and Kang, 2016). Existential threats are thought to elicit BIS activation because they are indicative of a conflict between current circumstances and existential needs; additionally, exposure to mortality and uncontrollability stimuli has been found to activate BIS-related brain systems (Gray and McNaughton, 2000; Klackl et al., 2018). Most individuals will engage in compensatory behaviors to manage existential threat (Hirsh and Kang, 2016; Trip et al., 2019), and individuals may become more receptive to radicalized ideology when exposed to existential threat that induces feelings of anxiety or uncertainty about their previously accepted identity, lifestyle, or beliefs (Wiktorowicz, 2005). Loss of control, a type of existential threat, has been found to induce an approach motivated state that then drives the pursuit of salient goals (Greenaway et al., 2015). Research on the uncertainty-identity theory (Hogg and Adelman, 2013) posits that it is when these feelings become pervasive that individuals are more strongly attracted to extremist groups, which can provide individuals a sense of identity and a strict architype for behavior, thus helping to reduce uncertainty from existential threat (Trip et al., 2019).

Furthermore, the BIS is also engaged during frustrative non-reward (Gray, 1987), causing individuals to experience persistent anxiety when they cannot reach their goals (Trip et al., 2019). Thus, commitment toward a new goal (*via* activation of the BAS) would aid in resolving conflict due to an inability to achieve other active goals, and extreme religious beliefs were associated with personal uncertainty enabled through the achieving an active goal (McGregor et al., 2013). In all, activity in the BIS increases feelings of anxiety in situations of heightened arousal and when prolonged (due to a highly active BIS), this increased anxiety can induce feelings of uncertainty, identity conflict, social avoidance, and decreased life satisfaction. This chronic BIS activation may then serve as a trigger for subsequent BAS activation (a pattern of reactive-approach motivation) and lead to an increase motivation to engage in defensive behaviors to mitigate stress and return to a state of equilibrium (Klackl et al., 2018; Trip et al., 2019).

Anxiety is the most direct trait correlate with heightened BIS sensitivity, and high BIS sensitivity is thought to increase propensity toward anxiety and other mood disorders (Hirsh and Kang, 2016). Individuals with heightened BIS sensitivity have also reported feeling less meaning and purpose in their lives (McGregor et al., 2013). Additionally, individuals with increased BIS sensitivity tend to be more attentive to the negative experiences and have more pessimistic outlooks (Hirsh and Kang, 2016). Anxiety about one’s identity, meaning, or significance in life are classified as existential threats, or threats to the existence of something—living or non-living (e.g., one’s self, country, or ideology). Sleegers et al. (2015) indicates that a variety of existential threats are associated with heightened activity in the areas of the brain related to BIS activation. Existential threats are thought to elicit BIS activation because they are indicative of a conflict between current circumstances and existential goals. The anxiety-to-approach model of threat and defense (Jonas et al., 2014) posits that individuals facing existential threat experience heightened anxiety and self-uncertainty due to increased BIS sensitivity and often employ reactive defensive strategies to escape these feelings (Trip et al., 2019).

Circumstances in which individuals perceive life as unfair or unjust—or experience various forms of deprivation—can induce feelings of uncertainty and anxiety due to the activation of the BIS (Trip et al., 2019). This perception of deprivation has been shown to induce motivation to restore feelings of significance (Webber and Kruglanski, 2018). This anxious quest for significance creates a unique opportunity for an ideology, or narrative to assign blame for these feelings to an external source or enemy, legitimizing feelings of aggression and supporting a concrete world view of what is “right” and “wrong” (Gøtzsche-Astrup, 2019).

Wiktorowicz (2005) also discusses how individuals may become more vulnerable and receptive to radicalized ideology when exposed to existential threat and begin to feel uncertain about their value or significance. It is when these feelings become pervasive that individuals are more strongly attracted to extremist groups (Jasko et al., 2017), which provide individuals a sense of identity and a strict prototype for behavior, helping to reduce uncertainty from existential threat and provide individuals with a sense of significance (McGregor et al., 2015; Trip et al., 2019). Further, uncertainty-identity theory supports that individuals are highly motivated to reduce feelings of uncertainty about their life, future, and identity (Hogg and Adelman, 2013). Current research, as well as research dating back to the 1930s, indicates that individuals more aggressively cling to external sources of identity to avoid anxiety from existential uncertainty (McGregor et al., 2015). These results are also in line with research indicating that BIS sensitivity positively predicts for both proactive and reactive aggression (Parker et al., 2020). High BIS sensitivity is associated with individual inclinations to states of anxiety, making persons more vulnerable to feelings of meaninglessness and uncertainty, and thus increasing vulnerability to the narratives of extremist or radical groups that prescribe a sense of significance and certainty (McGregor et al., 2015). In fact, it is the concrete ideas and values of radical groups that may attract high BIS types, as in-lab concreteness manipulations have been shown not only to reduce anxiety but also to increase goal-drive in various tasks (Watkins et al., 2008). Collectively, the findings on the role of BIS in reactive approach motivation (McGregor et al., 2015) as well as the known pathway to violent extremism through anxiety and a deprivation of identity/need fulfillment (Kruglanski et al., 2017) demonstrate a clear pathway to violent extremism in which exposure to violent extremist content online creates competing avoid/approach motivations which encourages the individual toward violent extremism to satisfy an anxiety-creating void.

## Discussion

### Future Empirical Assessment of the Reinforcement Sensitivity Theory-Four Motivational Pathways Model

It has been long argued that to move the psychological study of terrorism forward a multidimensional framework must be adopted that emphasizes the relationships between neural, cognitive, social processes, and behavior (Decety et al., 2018). Additionally, researchers are beginning to explore the neurological underpinnings of engagement in violent extremism (Pretus et al., 2018). The model provided above does not seek to replace current theoretical models of the radicalization process that involve personal, social, and cultural factors which influence an individual’s decision (e.g., Mink, 2015), but instead provide a common language (and underlying neurological pathway) through which this process can be conceptualized. In this sense, “vulnerability” to extremism represents the activation of a motivational pathway in response to exposure to extremist content.

Individuals who engage in extremist activity have, to varying degrees, and for varying functions, engaged with extremist material online (Gill et al., 2017). That said, existing research has not examined the psychological processes that underpin the interaction between the person, extremist propaganda, and any eventual extremist behavior (Reeve, 2019). The psychological processes underpinning online exposure and engaging in extremism are likely complex and dynamic (Horgan, 2019). One plausible way to overcome the stagnation in the psychological study of the terrorist is to apply lenses from more established fields of study (e.g., Ligon et al., 2015). Adopting such theoretical perspectives can have significant benefits, in that existing theories have much longer and more established empirical backing (Hunter et al., 2017). For example, psychologists were able to apply theories from Industrial/Organizational (I/O) psychology to terrorism by identifying the overlap between the challenges of traditional organizations and those of terrorist groups (e.g., recruitment or leader development; Hunter et al., 2017). Kruglanski et al. (2017) focused on the role of deviance and extremism (in general) in terrorist behavior to develop a general framework of violent extremism. In the current paper we focus on the role of motivation. One of the benefits of focusing on the RST is that it has a wealth of behavioral, psychological, neurological and physiological research that has established both the trait level individual differences associated with each form of motivation, and the state level physiological and neurological pathways that govern each motivational pathway. As such, the extant RST literature provides a framework to further study the model proposed above. Based on the RST model outlined above, we can hypothesize that which motivational pathway is activated (and indeed the intensity of that activation) is related to state and trait personality factors, as well as the phenomenological process of engaging with the material (which may be different based on the individuals mood, or the nature of the material; see Frischlich et al., 2015). Individuals for whom exposure to violent extremist content online create a *wanting* motivational state likely possess personality and neurological risk factors that predict intention to engage (i.e., testosterone-driven needs such as the need for social dominance and status) but also the ability to persist and strive to achieve a long-term goal which is associated with processes such as *delay-discounting*, and the ability to persist despite difficulties and fear (Howard and Crayne, 2019). In Table 1, we outline the overlap between correlates of the four proposed RST pathways with both personality factors associated with each type of response. Based on Table 1 we are able to put forward several hypotheses for future testing based on the presence of BAS/BIS associated traits (e.g., impulsiveness and sensation-seeking) and their reaction when exposed to extremist material (see Table 2). Furthermore, given that different BAS motivational reactions function independently, different patterns of BAS activation may occur based on an individual’s underlying BAS sensitivities. For example, individuals high in opioid associated traits such as reward sensitivity are more likely to have an approach-based *liking* response to exposure to extremist propaganda that is driven by liking (hedonistic pleasure) rather than a long-term behavioral desire to achieve a long-term goal (wanting). Thus, the specific nature of an individuals’ approach motivation in response to exposure to particular types of online extremist content can be predicted (e.g., avoidance vs., reactive approach vs., wanting vs., liking) based on the extent to which their baseline tendencies are associated with the correlates of each form of motivation. However, as “liking” often leads to “wanting,” it is also possible for individuals to be characterized by both high sensitivities in both BAS-wanting and BAS-liking and their associated personality traits (see Table 1) in which case an overlap in correlates for each motivational pathway would be expected. In these instances, it would be expected, regardless of the liking response, that if a wanting response is activated and the goal prioritized, an individual would be driven by incentive motivation to engage in a purposeful effort to achieve this goal.

**TABLE 1:** Reinforcement Sensitivity Theory (RST) pathways and associated personality traits and neurotransmitters.

Primary RST activation	Motivational state	Thematic facets	Personality correlates		Associated neurotransmitter
FFFS	Avoidance	Active Avoidance Flight Freeze	Neuroticism Anxiety Hostility Depression Self-consciousness Vulnerability Feelings	Reactive-aggression Volatility Fearfulness	Acetylcholine Adrenaline Noradrenaline
BIS	Reactive-approach motivation	Motor planning interruption Cautious risk assessment Obsessive thoughts Behavioral disengagement	Sensitivity to punishment *Neuroticism* Anxiety Hostility Depression Self-consciousness Vulnerability Agreeableness Straightforwardness	Altruism Compliance Modesty Tendermindedness Conscientiousness Order Dutifulness Deliberation	Cortisol GABA
BAS	Approach-liking	Reward responsiveness Reward reactivity	Hostility Extraversion Warmth Assertiveness Activity Positive affect Conscientiousness	Competence Order Dutifulness Achievement-striving Self-discipline Deliberation	Endogenous opioids
	Approach-wanting	Impulsivity Sensation seeking Reward interest	Sensitivity to reward *Neuroticism* Hostility Impulsiveness Vulnerability Extraversion Warmth Gregariousness	Activity Excitement-seeking Positive affect Openness Fantasy Actions Ideas Values	Testosterone Dopamine

*Thematic facets represent empirically derived constructs of RST per the RST-PQ (Corr and Cooper, 2016); personality correlates were those defined by the NEO-PI-R (see Keiser and Ross, 2011; Segarra et al., 2014); associated neurotransmitters for the BAS are reported according to Krupić and Corr (2017), for the BIS, neurotransmitters are reported according to Tops and Boksem (2011) and Edden et al. (2012); for the FFFS neurotransmitters are reported according to Roelofs (2017).*

**TABLE 2:** Possible hypotheses involving Reinforcement Sensitivity Theory (RST) personality traits and reactions following exposure to extremist content.

Primary RST activation	Possible hypotheses regarding reactions to extremist content	Possible hypotheses regarding personality	Possible hypotheses regarding cognitive outcome
FFFS	Individuals with increased FFFS sensitivity would be expected to be more likely avoid, or not engage with extremist content.	Individuals with increased FFFS sensitivity would be expected to: Have increased scores for personality measures related to the Neuroticism domain of the NEO-PI-R listed in Table 1. Have decreased scores for personality measures related to Action, or goal-perusal.	Individuals with increased FFFS sensitivity would be expected to report lower scores on known scales associated with the measurement of extremist intent and extremist cognitions.
BIS	Individuals with increased BIS sensitivity would be expected to experience reactive approach motivation and experience subsequent activation of the BAS when exposed to extremist content, and thus be more likely to engage with extremist content.	Individuals with increased BIS sensitivity would be expected to: Have increased scores for personality measures related to Anxiety and the Neuroticism, Agreeableness, and Conscientiousness domains of the NEO-PI-R listed in Table 1. Have decreased scores for personality measures related to Extraversion, Action, and Positive Affect.	Individuals with increased BIS sensitivity would be expected to have increased vulnerability to the goals and narratives of extremist or radical groups and thus, report increased scores on known scales associated with the measurement of extremist intent and extremist cognitions.
BAS	Individuals with increased BAS-liking sensitivity would be expected to experience approach motivation and be more likely to experience hedonic pleasure from engagement with extremist content, and thus be more likely to further engage with extremist content.	Individuals with increased BAS sensitivity related to Reward Responsiveness and Reward Reactivity would be expected to: Have increased scores for personality measures related to Hostility and the Extraversion, and Conscientiousness domains of the NEO-PI-R listed in Table 1. Have decreased scores for personality measures related to sensitivity toward punishment and deliberation.	Individuals with increased BAS-liking sensitivity would be expected to gain a hedonistic pleasure from engagement with extremist materials, and report increased positive responses to extremist media, but with no concurrent activation of a motivational state to engage with the goals being depicted in the media, and thus report decreased, or average scores on known scales associated with the measurement of extremist intent and extremist cognitions.
	Individuals with increased BAS-wanting sensitivity would be expected to experience incentive motivation and thus, be more likely to engage with extremist content.	Individuals with increased BAS sensitivity related to Impulsivity, Sensation Seeking, and Reward Interest would be expected to: Have increased scores for personality measures related to Hostility, Impulsivity, and the Neuroticism, Extraversion, and Openness domains of the NEO-PI-R listed in Table 1. Have decreased scores for personality measures related to sensitivity toward punishment and the Agreeableness and Conscientiousness domains of the NEO-PI-R.	Individuals with increased BAS-wanting sensitivity would be expected to experience activation of a motivational state to engage with the goals being depicted in the media and report higher scores on known scales associated with the measurement of extremist intent and extremist cognitions.

### Limitations

While the proposed pathway model provides several important benefits to the psychological study of violent extremism, there are several important limitations to consider. The first of those is that this paper does not adopt incorporate all processes that exist within the RST framework and indeed offer even more theoretical granularity as it relates to motivation. In a review of the five most frequently used RST questionnaires, Krupić et al. (2016) classified the BAS scales from the five questionnaires into four groups that represented different forms of BAS activation: wanting, striving, liking, and capturing. Despite the distinction of wanting/linking being the more established BAS separation, with current neuroscience studies not clearly disaggregated “wanting” and “incentive motivation” (e.g., Berridge and Robinson, 2003, 2016), there is an important difference within “wanting” between wishing for something (wanting) and acting to attain it (striving/capturing). Furthermore, these four BAS processes can also be viewed as stages within a single longitudinal dynamic process in which a single individual moves throughout a dynamic BAS process that begins with wanting, moves through striving, to capturing and finally liking (Corr and Krupić, 2017). Thus, within the BAS system, there is a spatial-temporal dynamic in which an individual moves through different stages BAS, rather than being differentiated across different BAS processes. In this article, we focus on the initial exposure to extremist content and the implications of four different forms of motivational reaction (avoidance, reactive-approach, approach-wanting, and approach-liking), that said the journey beyond first exposure that one takes is likely to be far more complex and potentially represent the longitudinal BAS process proposed by Krupić and Corr (2017) that include the striving and capturing aspects of the BAS not covered in the current paper. Thus, while this paper outlines what could be perceived as a first-contact typology of reactions, the longitudinal process that governs the movement toward behavior could involve multiple RST pathways working in tandem, or even in parallel. This while this paper proposes a typology of RST reactions, and emerging researching is supporting the role of BAS processes in governing reactions to extremist content (Shortland and McGarry, 2022), more research is needed to fully understand the complex role that RST processes play in the movement toward terrorist behavior.

Despite these limitations, recent reviews of terrorism research have argued that the field is no closer to answering the simple question, “What leads a person to turn to political violence?” than it was 10 years ago (Sageman, 2014). Other reviews, while less extreme in their conclusion, do maintain that the field requires better empirical assessment. Silke (2013) argues that more needs to be done before building on past research, rather than just rehashing it (p. 34). Schuurman’s (2019) analysis of over 3,000 articles published in leading terrorism-specific journals between 2007 and 2016 found that over half used primary sources. But in terms of psychological theory, most theories remain unsubstantiated and unfalsifiable, relying on metaphors such as pathways, stairways, and pyramids. Thus, this provides an opportunity for falsifiable hypotheses to be developed, tested, and if appropriate, rejected. This remains the cornerstone of the scientific method yet has long eluded those who study terrorism (Lygre et al., 2011). To put this point in perspective, we present in Table 2 a series of testable and falsifiable hypotheses using common RST principles/methods (Corr and McNaughton, 2012; Gerson et al., 2017; Bacon et al., 2018; Satchell et al., 2018). As can be seen in Table 2, a series of hypotheses involving personality traits (i.e., hostility) and reaction to exposure to extremist material.

In addition to this, while one of the important benefits of disaggregating the motivations of those who view extremist material online is the ability to infer judgments of risk, there is no *a priori* evidence that a liking reaction *de facto* implies lower risk. Instead, it could imply greater risk. For example, it is viable that the short-term hedonistic pleasure associated with the concept of liking could also be used to explain the decision for a violent extremist offender to engage in a short time-frame attack that involves little (to no) planning such as crude knife, vehicle, or firearm attacks we have seen in recent years. What this means then is that while we may differentiate viewer motivation, the liking/wanting differentiation does not necessarily dissociate those who do act, from those who do not. Instead, it may differentiate those who engage in a long-term planning process, vs. a short-term planning process. Again, this does not invalidate the need nor theoretical importance of RST in the study of violent extremism, but it does limit the conflation of differentiation of process and of outcome.

## Conclusion

It has long been argued that in order to move the psychological study of terrorism forward a multidimensional framework must be adopted that emphasizes the relationships between neural, cognitive, and social processes (Decety et al., 2018). In this paper, we conceptualized the effects of exposure to extremist content online *via* the RST model of motivation. This model does not seek to replace current theoretical models of the radicalization process that involve personal, social and cultural factors influencing an individual’s decision (e.g., Mink, 2015), but instead provides a common language (and underlying pathways) through which this process can be conceptualized. Specifically, many of the processes conceptualized within the RST model map onto previous assertions about the process of radicalization. In most, there is a general fear reaction from exposure to violent extremism (conceptualized here as FFFS-driven avoidance; Shortland et al., 2017). In some, violent extremism provides an avenue to address underlying anxieties about oneself and their identify (reactive-approach and the quest for significance; McGregor et al., 2015; Kruglanski et al., 2017). Others are drawn to extremist content due to a range of BAS-driven correlates such as sensation-seeking, a sense of adventure, or the “thrill” and “excitement” (Haggerty and Bucerius, 2020). Finally, in those who are drawn to such content, there are those whose pleasure comes from simply viewing and engaging with extremist content (Shortland, 2016) and those whose engagement drives a long-term motivational goal to engage with violent extremist content or action (liking vs. wanting; see Berridge and Robinson, 2003).

We agree that no one theory is a panacea of violent extremism. However, the current state of the research studying violent extremist content online is missing is validated empirical frameworks that can be used to guide empirical testing of hypotheses and thus, the advancement of theory (Conway, 2017, p. 82; Frissen, 2021). RST is consistently used as a motivational framework to explain the effect of wider personality, social and environmental influences on motivational behavior. Furthermore, those who have used RST as a framework to explore a range of behaviors (both pro and anti-social) have identified that different forms of behavior are associated with different forms of BAS activation. Thus, while the outlined model is theoretical, and indeed the hypotheses are exploratory, each is based on a wealth of literature that has previously explored the relationships among personality, neurological processing, motivation, and behavioral outcomes. Furthermore, each can be tested through standard methods used by psychologists to study the role of neurological and physiological predictors of reactions to media content (e.g., Bushman, 1995; Carnagey et al., 2007; Zillmann and Weaver, 2007) and, critically, these hypotheses are falsifiable, allowing this model to be refined, validated, or rejected.

## Data Availability Statement

The original contributions presented in the study are included in the article/supplementary material, further inquiries can be directed to the corresponding author.

## Author Contributions

NS and PM developed the theoretical framework for the manuscript. JP led the investigation and outline of perspectives from biosocial criminology. AP, TG, and NS provided review and expert insight for theory development. All authors contributed to the article and approved the submitted version.

## Conflict of Interest

The authors declare that the research was conducted in the absence of any commercial or financial relationships that could be construed as a potential conflict of interest.

## Publisher’s Note

All claims expressed in this article are solely those of the authors and do not necessarily represent those of their affiliated organizations, or those of the publisher, the editors and the reviewers. Any product that may be evaluated in this article, or claim that may be made by its manufacturer, is not guaranteed or endorsed by the publisher.

## Footnotes

1 Throughout this paper when we refer to violent extremist content online we follow the concept of “terroristic content” in that it legitimizes, motivates or enables acts of violence against civilian targets (Holbrook et al., 2013).
